# Intervention development to reduce sedentary behaviour among adults: a qualitative investigation using the Behaviour Change Wheel

**DOI:** 10.1186/s12966-026-01917-w

**Published:** 2026-04-21

**Authors:** Hannah Harsanyi, Nicole Slot, Reilly Parkinson, Andria R. Morielli, Apiramy Jeyapalan, Elizabeth Holmes, Susan Flynn, Tavis Campbell, Hude Quan, Yong Zeng, Jiami Yang, Shaminder Singh, Jennifer Vena, Stuart Biddle, Christine M. Friedenreich, Lin Yang

**Affiliations:** 1 Department of Cancer Epidemiology and Prevention Research, Cancer Care Alberta, Arthur Child Cancer Centre, 3395 Hospital Drive NW, Calgary, AB T2N 5G2 Canada; 2 Department of Cancer Prevention and Screening Innovation, Primary Care Alberta, Southport Tower, 10301 Southport Ln SW, Calgary, AB T2W 3N2 Canada; 3 Canadian Cancer Society, 55 St Clair Avenue West, Suite 300, Toronto, ON M4V 2Y7 Canada; 4 Department of Psychology, University of Calgary, 2500 University Dr NW, Calgary, AB T2N 1N4 Canada; 5 Department of Oncology, Cumming School of Medicine , University of Calgary, 3330 Hospital Drive NW, Calgary, AB T2N 4N1 Canada; 6 Department of Community Health Sciences, Cumming School of Medicine, University of Calgary, 3330 Hospital Drive NW, Calgary, AB T2N 4N1 Canada; 7 Gina Cody School of Engineering and Computer Science, Department of Cybersecurity and Intelligent Systems Engineering, Concordia University, 1455 De Maisonneuve Blvd. W, Montreal, QC H3G 1M8 Canada; 8 Faculty of Health, Community and Education, School of Nursing and Midwifery, Mount Royal University, 4825 Mount Royal Gate SW, Calgary, AB T3E 6K6 Canada; 9 Alberta’s Tomorrow Project, Cancer Care Alberta, 3395 Hospital Drive NW, Calgary, AB T2N 5G2 Canada; 10 Institute for Health, University of Southern Queensland, University Drive, Education City, 37 Sinnathamby Blvd., Springfield Central, QLD 4300 Australia

**Keywords:** Sedentary Behaviour, Sitting Time, Lifestyle Factors, Cancer Prevention, Risk Reduction, Behaviour Change, Qualitative Research

## Abstract

**Background:**

Reducing prolonged sedentary behaviour is recommended to improve population health. The development of behaviour change interventions requires understanding context-specific determinants, which may differ across domains of transportation, occupational, and leisure time sedentary behaviour. This study aimed to use the Behaviour Change Wheel to inform the development of domain-specific intervention strategies for reducing prolonged sedentary behaviour among Canadian adults.

**Methods:**

One-on-one semi-structured interviews were conducted with a diverse sample of adult participants from across Canada. Determinants of reducing prolonged sedentary behaviour were identified through thematic analysis and characterized according to their relevance to domains of transportation, occupational, and leisure time sedentary behaviour. The Behaviour Change Wheel and Behaviour Change Techniques Taxonomy (BCTTv1) were used to identify intervention strategies, components, and modes of delivery specific to and across behavioural domains.

**Results:**

Thirty participants were interviewed (63% women, 60% age 25–44, and 50% identified as white). While all participants endorsed potential benefits of and personal goals related to reducing prolonged sedentary time, many had difficulty implementing these changes in their day-to-day life. Most participants identified leisure time as the domain in which they were most keen to reduce prolonged sedentary time. Barriers to reducing sedentary behaviour varied by domain: transportation-related barriers were primarily linked to physical opportunity, occupational barriers to social opportunity, and leisure time barriers to automatic motivation. Intervention techniques including prompts/cues, self-monitoring, and goal setting were identified to overcome barriers to reducing sedentary behaviour across domains. Restructuring the physical and social environment were identified as being particularly significant for enabling behaviour change in transportation and occupational domains, respectively.

**Conclusions:**

Individual-level interventions, such as goal-oriented prompts or cues, may be well-suited to reducing leisure time sedentary behaviour. In contrast, multilevel interventions, including organizational social restructuring and urban planning policy changes may be needed to address significant opportunity-related barriers in occupational and transportation domains.

**Supplementary Information:**

The online version contains supplementary material available at 10.1186/s12966-026-01917-w.

## Background

Sedentary behaviour is defined as “any waking behaviour characterized by an energy expenditure ≤ 1.5 metabolic equivalents, while in a sitting, reclining or lying posture” [1]. Given technological and cultural changes in recent years, time spent on sedentary behaviour, particularly related to screen use, is high and rising [2, 3]. Estimates of sedentary behaviour vary by measurement approach, with self-reported data suggesting over 5 h per day and accelerometer data indicating more than 9 h per day among Canadian adults [3, 4]. Similarly, high levels of daily sedentary behaviour are reported globally, with estimates exceeding 6 h per day among American adults based on self-reported data [2] and ranging from 2.5 to 10 h per day among European adults across measurement approaches, studies, and countries [5].

High levels of sedentary behaviour pose a significant public health concern, as prolonged sedentary behaviour has been associated with increased risk of several adverse health outcomes, including cancer, diabetes, cardiovascular disease, and all-cause mortality [6–8]. In 2020, the World Health Organization (WHO) guidelines on physical activity were updated and for the first time incorporated general recommendations to limit sedentary behaviour [9]. Canada is one of the first countries to provide quantified targets for the amount of sedentary time, with the 24-Hour Movement Guidelines recommending adults limit daily sedentary time to 8 h or less, including no more than 3 h of recreational screen time [10].

Despite the recognized need to modify sedentary behaviour, there is limited evidence on how best to accomplish this change, with particular gaps in identifying interventions that can generate long term changes in behaviour [11, 12]. The design of interventions targeting sedentary behaviour is particularly complicated since influencing factors differ significantly based on the type and context of the behaviour [13]. For example, the determinants of spending time driving are likely quite different compared to time spent on the computer, and each of these behaviours may be additionally affected by their context (e.g., occurring while at work vs. during free time). Therefore, interventions that systematically consider and target domain-specific determinants of sedentary behaviour are likely needed to make meaningful changes in sedentary time [14]. However, few studies to date have systematically analyzed these factors when designing interventions to reduce sedentary behaviour.

The systematic design of targeted interventions can be facilitated by the Behaviour Change Wheel (BCW), which offers a comprehensive theory-based framework to guide the development of behaviour change interventions and policies [15, 16]. At the core of the BCW, the COM-B model considers relationships among sources of behaviours, i.e., external/contextual factors [opportunity (O)] and individual factors [capability (C) and motivation (M)] which determine behaviour (B), either facilitating or inhibiting the target behaviour [16]. By considering these interrelated sources of behaviour, the BCW allows for tailored intervention development based on the type and context of the target behaviour. Using this framework, our study aimed to characterize determinants of prolonged sedentary behaviour across domains of transportation, occupational and leisure time sedentary behaviour, and identify domain-specific intervention strategies for reducing prolonged sedentary behaviour among Canadian adults.

## Methods

### Recruitment

Participants were recruited through collaboration with the Canadian Cancer Society (CCS), which is a national charitable organization providing funding, resources, and advocacy for cancer prevention initiatives across the country. Study recruitment materials were sent to potential participants through a CCS newsletter, via email to CCS volunteers and registered users of their online cancer prevention tool [17], as well as through posts on the CCS website and social media accounts. Interested individuals completed a screening questionnaire that collected information about their place of residence, sociodemographic characteristics, and patterns of sedentary behaviour. Eligible participants were 25 years or older, living in Canada, owned a smartphone, and identified themselves as sitting for an average of 6 or more hours per day. Exclusion of individuals under age 25 was selected to reduce heterogeneity associated with social and biological transitions of emerging adulthood [18, 19]. A cutoff of 6 h per day of sedentary behaviour was used to reflect the dose-response risk accumulation pattern reported in studies associating sedentary time with health outcomes [7, 8, 20], as well as the average self-reported sedentary behaviour among Canadian adults [4].

Responses to the screening questionnaire were used to purposively sample interview participants in accordance with a recruitment framework developed by the CCS and designed to ensure diversity across key sociodemographic characteristics in the Canadian population. This framework was used to establish recruitment targets across geographical regions, rural places of residence, age groups, racial or ethnic backgrounds (including consideration of Indigenous identities), gender identities, sexual orientations, income levels, and residency statuses (Supplementary Material 1). An initial sample size of 30 participants was selected based on established qualitative research practice, balancing sufficient breadth to capture variation across these characteristics with depth of inquiry required for theory-informed analysis [21–23]. This sample size was considered appropriate for identifying recurrent themes and domain-specific determinants across sources of behaviour, with flexibility for additional recruitment if data saturation was not achieved. Concurrent analysis of interviews was conducted to assess data saturation, which was identified by lack of emergence of new domain-specific determinants in consecutive interviews.

### Data collection

Purposively selected participants provided informed consent and completed a one-on-one semi-structured interview to understand their knowledge about, patterns of, and barriers to reducing prolonged sedentary behaviour. The interview guide was developed with questions related to each domain of sedentary behaviour (transportation, occupational, and leisure time) as well as sources of behaviour from the COM-B model (capability, opportunity, and motivation) (Supplementary Material 2). The transportation domain referred to time spent travelling, including commuting to and from work or other locations, such as the grocery store; the occupational domain referred to time spent completing paid work, volunteering, attending classes, and/or studying; and the leisure time domain referred to all time spent outside of transportation or occupational domains, including recreational screentime, mealtimes, hobbies, household duties, etc. Within interviews, the term ‘sitting time’ was used in place of ‘sedentary behaviour’ and ‘prolonged’ was indicated by phrases such as ‘long periods’, thereby functioning as a qualitative descriptor rather than a defined amount of time. These terminological choices were designed to increase interpretability and ease of communication within interviews, which were all conducted among ambulatory adults.

Interviews were conducted virtually via Microsoft Teams between February-September 2023 (R.P.) and ranged from 15 to 45 min in length. Interview content varied according to participants’ self-reported sedentary behaviour patterns, with interview sections and questions selected by the interviewer based on topics identified by participants as relevant to their personal experiences [24]. Interviews were recorded and machine transcribed; transcripts were cleaned by study investigators (H.H. & N.S.) to ensure accuracy and inclusion of participants’ nonverbal responses or cues. Participants were assigned a unique study identification number and names or other identifying information were removed from transcripts to enable participant anonymity. Participants were offered a CAD $50 gift card for their time and participation.

### Data analysis

**Fig. 1:**
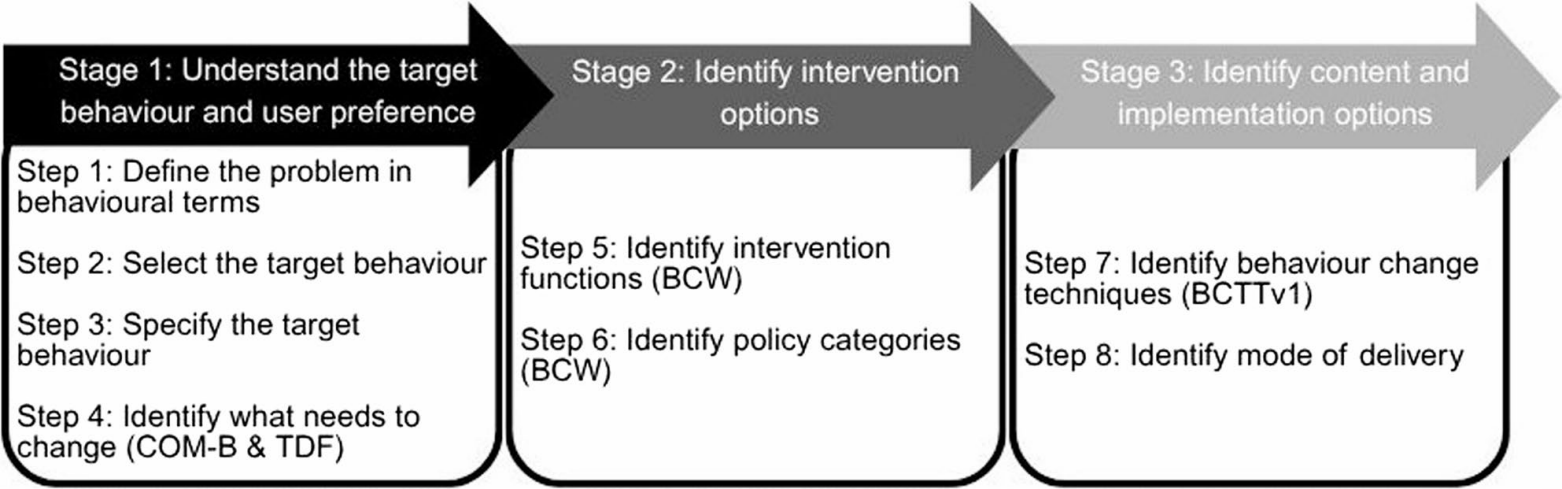
Stages of Intervention Development guided by the Behaviour Change Wheel (BCW). Abbreviations: BCW – Behaviour Change Wheel; BCTTv1: Behaviour Change Technique Taxonomy, version 1; COM-B – Capability, Opportunity, Motivation- Behaviour model; TDF – Theoretical Domains Framework

**Fig. 2:**
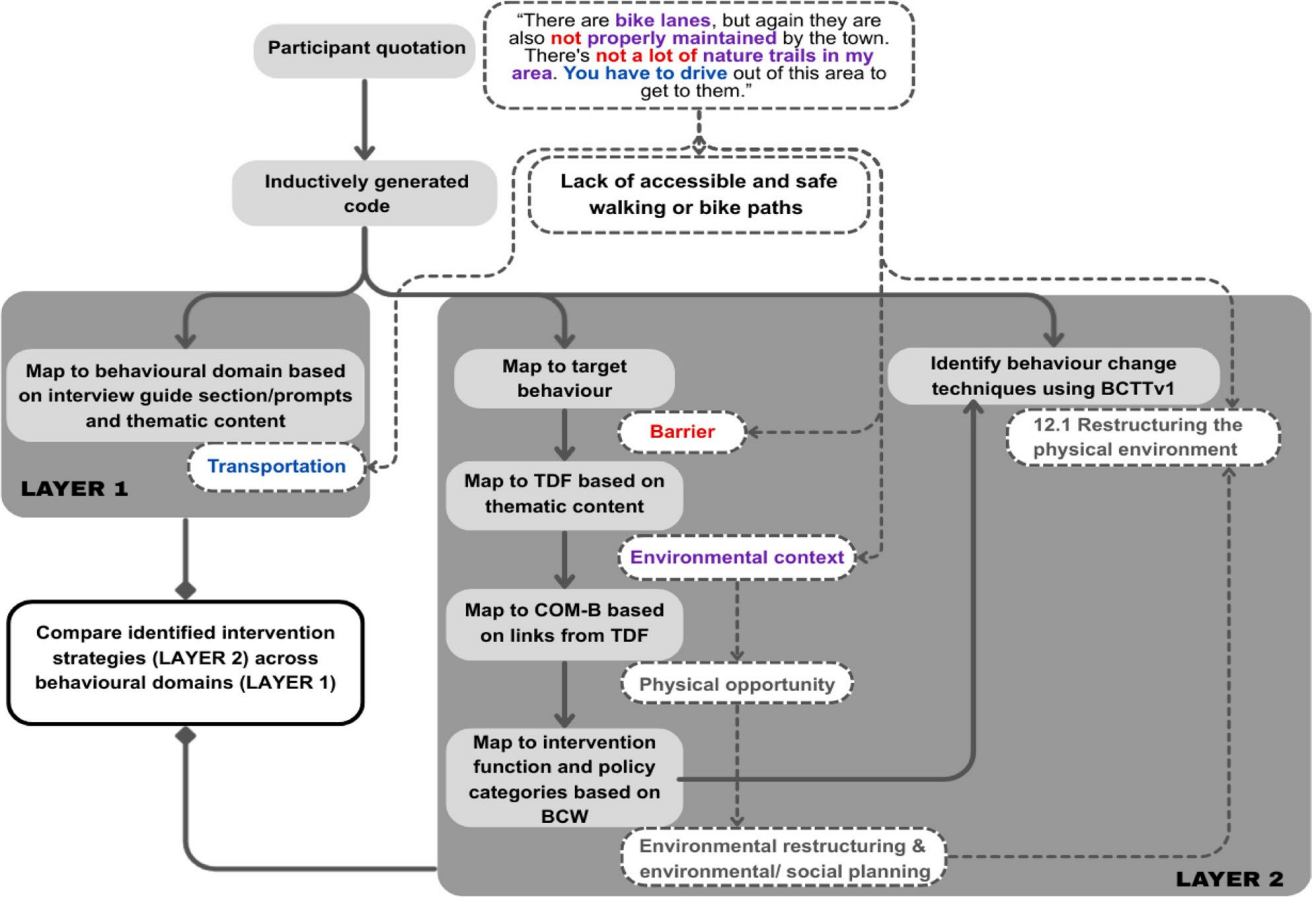
Analytical process map depicting the analytical workflow (solid path) and the parallel analysis of an example quotation (dashed path). Additional example quotations for each determinant are provided in Supplementary Material 3, with the example provided above available in Table A3.2, row 3. Abbreviations: BCW – Behaviour Change Wheel; BCTTv1: Behaviour Change Technique Taxonomy, version 1; COM-B – Capability, Opportunity, Motivation- Behaviour model; TDF – Theoretical Domains Framework

A hybrid inductive-deductive approach was used to analyze data from individual semi-structured interviews. Inductive coding was conducted by applying a qualitative descriptive approach with thematic analysis [25, 26]. Data familiarization was undertaken by repeated listening to the interview recordings and reading of transcripts. Detailed notes were taken to capture preliminary impressions of thematic content across interviews. Two researchers (HH & NS) independently coded interviews using NVivo software, with weekly discussions regarding reflexivity and coding decisions to facilitate iterative coding and consistency between researchers. The inductively generated codes were subsequently mapped to the BCW following a deductive process informed by the steps of intervention development as outlined in Fig. 1 and described below [15, 16]. Deductive coding was principally conducted by HH with collaborative input and verification by NS; any discrepancies were resolved through discussion and consensus. The overall analytical process is depicted in Fig. 2.

#### Steps 1 & 2

To ground the analysis, the problem was defined as the need to reduce high levels of prolonged sedentary behaviour among adults to improve health outcomes and facilitate disease prevention in Canada. Therefore, the target behaviour was defined as reducing prolonged sedentary behaviour across transportation, occupational, and leisure time domains.

#### Step 3

Details on the type and domain of sedentary behaviour as described within qualitative interviews were used to characterize the relevance of each code to behavioural domains. Each code was categorized as being either domain-specific (with relevance exclusively to transportation, occupational, or leisure time sedentary behaviour) or general (relating to multiple domains).

#### Step 4

Codes were categorized as barriers or facilitators to the target behaviour, i.e., enabling or inhibiting a reduction in prolonged sedentary behaviour. These determinants were mapped to the Theoretical Domains Framework (TDF) which was used to identify relevant sources of behaviour from the COM-B model [27].

#### Steps 5 & 6

Intervention functions and policy categories were identified from the BCW for each determinant [16]. Links between COM-B components and intervention functions were used to identify intervention function(s) for barriers in which the source of behaviour was assessed as being absent (i.e., when participants were lacking capability, opportunity, or motivation). Linking or non-linking intervention functions were identified to leverage facilitators or to work around barriers when the COM-B source of behaviour was assessed as being partially present or achieved.

#### Steps 7 & 8

Each determinant was mapped to the Behaviour Change Technique Taxonomy (BCTTv1) to identify recommended content of interventions with reference to participant quotations captured within the code [28]. The recommended mode of delivery specific to each technique was described in a narrative synthesis of themes arising from the qualitative interviews.

A detailed audit trail of coding decisions was kept throughout the analysis. Triangulation was conducted by comparing data familiarization notes with inductively generated codes to ensure overarching impressions of participant perspectives were captured in the analysis. Quotations were selected verbatim from the transcripts to provide supporting information for identified themes; repeated or filler words were removed and author insertions to provide missing contextual information are indicated in quotations using square brackets.

## Results

### Participant characteristics

A total of 815 individuals completed the online screening questionnaire, of which 593 met inclusion criteria and 30 were purposively selected and subsequently completed a semi-structured qualitative interview. Participants from across Canada were included, most were Canadian citizens (87%), and 20% lived in settings with population < 50,000. The study sample included participants of diverse racial/ethnic backgrounds (including those of Indigenous identities), sexual orientations, and gender identities (Table 1). Most participants were women (63%), 25–44 years old (60%), identified as white or European racial/ethnic background (50%), and reported low-to-middle personal income level (63% <$80,000CAD).

**Table 1:** Sociodemographic Characteristics of Participants (*n* = 30)

Sociodemographic Variable	Number of Participants (%)
Canadian Region	
Atlantic region (PEI, NL, NB, NS)	4 (13.3%)
Prairie region (AB, SK, MB)	10 (33.3%)
Quebec	2 (6.7%)
Ontario	9 (30.0%)
British Columbia	5 (16.7%)
Rurality*	
Metropolitan centre (population (pop.) > 1 million)	12 (40.0%)
City (pop. 250,000–1 million)	6 (20.0%)
Town (pop. 50,000-250,000)	5 (16.7%)
Rural (pop. <50,000)	6 (20.0%)
Prefer not to say	1 (3.3%)
Residency Status	
Canadian Citizen	26 (86.7%)
Permanent Resident	3 (10.0%)
Prefer not to say	1 (3.3%)
Age Group	
25 to 44	18 (60.0%)
45 to 64	9 (30.0%)
65+	3 (10.0%)
Racial/Ethnic Background	
White or European	15 (50.0%)
Chinese or other East Asian	5 (16.7%)
Filipino, South Asian, or West Asian	5 (16.7%)
Black	1 (3.3%)
First Nations	1 (3.3%)
Latin American	1 (3.3%)
Mixed (multiple selected options)	2 (6.7%)
Gender Identity	
Woman	19 (63.3%)
Man	10 (33.3%)
Non-binary or other gender	1 (3.3%)
Sexual Orientation	
Heterosexual or straight	21 (70.0%)
Bisexual	5 (16.7%)
Gay or lesbian	4 (13.3%)
Personal Annual Income (CAD)	
0 to $39,999	8 (26.7%)
$40,000 to $79,999	11 (36.7%)
$80,000 to 119,999	8 (26.7%)
$120,000 or more	1 (3.3%)
Prefer not to say	2 (6.7%)

*Rurality categorizations were based on CCS definitions at the time of data collection which have since been updated to define rural communities as those with a population of < 10,000

### Behavioural determinants of reducing sedentary behaviour

We identified 20 barriers and 14 facilitators to reducing prolonged sedentary behaviour, which were mapped to all sources of behaviour from the COM-B model (Table 2). While a range of domain-specific determinants of reducing prolonged sedentary behaviour were identified, nearly half of determinants (16/34) were applicable across domains. Importantly, leisure time was identified by most participants as the domain in which they were most keen to reduce prolonged sedentary time. Consequently, the leisure time domain corresponded to the largest number of domain-specific determinants of reducing prolonged sedentary behaviour (9/18).

**Table 2:** Summary of barriers and facilitators to reducing prolonged sedentary behaviour (SB), mapped to sources of behaviour from the COM-B model

COM-B Component	Barriers	Facilitators
Psychological Capability	Lack of specific knowledge regarding SB guidelines^†^; Lack of specific knowledge on health benefits^†^; Difficulties motivating self or breaking existing habits^†^	Awareness that sedentary lifestyle can have negative health impacts^†^
Physical Capability	Limitations or injuries may make it difficult to break up SB^†^	Physically able to engage in non-sedentary activities^†^
Social Opportunity	Social isolation^◊^; Shared interests with friends/family in TV, video games, or virtual activities^◊^	Interacting with friends, family, and co-workers^†^; Workplace culture that encourages breaks^§^
Physical Opportunity	Poor weather and shorter days in the wintertime^†^; Significant distance between home and common destinations (amenities or work) ^‡^; Limited access to public transportation, particularly in small communities^‡^; Lack of accessible and safe walking or bike paths^‡^; Widespread access to technology^◊^	Creating physical distance between self and objects^†^; Workplace resources to help decrease SB, such as a standing desk^§^; Increased flexibility and autonomy working from home^§^
Automatic Motivation	Cyclical relationship between poor mood and increased SB^†^; Feeling drained or tired^†^; Engrained habits regarding driving^‡^; Routine involves ‘winding down’ in the evenings^◊^; Screentime activities are addicting and easy to get lost in^◊^; Sedentary activities are relaxing and can be a form of escape^◊^; Sedentary activities can be rewarding by offering mental engagement or stimulation^◊^	Reminders to break up sedentary time^†^; Extended sedentary time causes stiffness or soreness^†^; Participating in active, non-sedentary hobbies can be rewarding and feels good^◊^
Reflective Motivation	Challenges keeping up with expectations at work^§^; Need to focus for extended periods or ‘get in the zone’ to accomplish occupational tasks^§^	Desire to reduce amount of sedentary time^†^; Desire to reduce screentime^†^; General belief that being less sedentary would benefit short-term and long-term health^†^; Responsibilities, such as taking care of children, pets, or household duties, encourages greater activity^◊^

^†^ Relevant to general sedentary behaviour, across domains

^‡^ Relevant to transportation sedentary behaviour

^§^ Relevant to occupational sedentary behaviour

^◊^ Relevant to leisure time sedentary behaviour

#### Capability

Capability-related determinants were relevant across behavioural domains, being related to participants’ assessment of their abilities and capacity. Although a few participants expressed barriers related to their physical capability, such as injuries or comorbidities, most participants endorsed having the physical ability to reduce prolonged sedentary behaviour. Most references to capability-related barriers were psychological, with themes related to knowledge and behavioural regulation.

*“I could easily go for a walk*,* but it’s just I would be walking by myself. There’s less onus on me to do it because it would be out of my own self-discipline.”*

Participant 233, woman, from large city in Alberta.

Notably, while participants expressed a general understanding that prolonged sedentary behaviour could negatively impact their health, they lacked specific knowledge regarding guidelines or estimates of how much sedentary time would be “too much”. Furthermore, participants often connected their understanding of sedentary behaviour with guidelines for physical activity or exercise, rather than differentiating the two concepts.

*"I haven’t heard about sitting time. Just everybody says you need to get some exercise."*

Participant 451, man from rural Saskatchewan.

#### Opportunity

Opportunity was seen as influencing sedentary behaviour across domains, however was most predominantly discussed when referring to transportation sedentary behaviour. Barriers to engaging in active rather than sedentary transportation were primarily related to environmental context, as participants highlighted needing to travel long distances, having unsatisfactory access to public transportation, and limited infrastructure that enabled biking and/or walking. These barriers were particularly highlighted among participants who lived in rural areas and were also largely influenced by the season and weather.

*"During wintertime, it’s awful for me, I’m going to be lazy… I don’t have any urge to go outside."*

Participant 628, man from town in Ontario.

*"I live in a small community, there’s only a convenience store here, so I wouldn’t do my grocery shopping there. I have to go to the next town."*

Participant 053, woman from rural British Columbia.

In the occupational domain, prolonged sedentary behaviour was largely influenced by determinants related to workplace culture, social interactions with co-workers, and resources, such as standing desks or other components of office organization. Notably, several participants reflected on differences resulting from working remotely after the COVID-19 pandemic. Although the theme of greater flexibility and autonomy in the home environment was identified as a facilitator to reducing prolonged occupational sedentary behaviour, many participants also felt they had fewer opportunities to connect with co-workers and were thereby less likely to break up their sedentary behaviour. As a result, the perceived impact of working from home on sedentary behaviour was mixed, with differences influenced by participants’ individual assessment of home or office organization, social influences, and work-related demands.

*"It’s a little easier when I’m at home to maybe [take breaks], go empty the dishwasher or whatever, throw a load of laundry in."*

Participant 684, woman from rural Ontario.

*"When you’re in the office things are farther from you…so the tendency to walk longer is definitely more [and] you definitely will have more distraction because people may come to you, ask questions or you may need to go to their cubicle, discuss something… this actually causes you to get off your seat."*

Participant 160, man from metropolitan centre in Ontario.

Across domains, social opportunity was identified as an important facilitator to reducing prolonged sedentary behaviour, as social influences were generally seen as helpful for encouraging reductions in sedentary behaviour and social isolation was often seen as contributing to prolonged sedentary behaviour. Although this impacted several participants with differing residency statuses, it was particularly emphasized among immigrants or individuals who moved to new or unfamiliar communities.

*"Being an immigrant, it’s gonna be like everything is new… I don’t have the friends from back home, so it’s gonna be a totally different scenario. Yeah, that makes a huge impact [on my sedentary behaviour]."*

Participant 628, man from town in Ontario.

Although social influences were more commonly associated with facilitating a reduction in sedentary behaviour, in the leisure time domain, social connections with shared interests in sedentary activities, particularly interests related to screentime, were seen as hindering reductions in prolonged sedentary behaviour.

*"Everyone after meals sort of resorts to screens, so then I’ll just resort back to my room to study or to watch some videos. So, what everyone else is doing kind of leaves me to do the same thing and that I feel influences my behaviors definitely."*

Participant 233, woman from city in Alberta.

#### Motivation

Motivational barriers to reducing prolonged sedentary behaviour were most predominant within the leisure time domain. In this domain, the enjoyable and, at times, addictive features of sedentary activities acted as automatic motivation which inhibited participants from reducing prolonged sedentary behaviour.

*"I just get sucked into a show and keep wanting to watch the next episode. Sometimes there will be times where I really need to use the washroom, but this show is too good and I won’t even get up to do that."*

Participant 173, woman from rural New Brunswick.

In the occupational domain, motivation was identified as a barrier in the reflective, rather than automatic dimension, as participants expressed limitations related to their professional role. Most participants (73%) were employed or engaged in primarily sedentary occupations where they prioritized achieving professional goals or meeting expectations which inhibited their ability to break up prolonged sedentary behaviour.

*"[My work] requires getting into things. So, it’s very difficult to just do something for half an hour… I have a tendency to not want to break things up because I lose my train of thought."*

Participant 492, woman from metropolitan centre in Quebec.

Across behavioural domains, participants reflected on how emotion influenced their motivation, as poor mood and tiredness led to decreased motivation to break up or reduce sedentary time.

*"It’s like a negative feedback loop. The more I sit down, the more grumpy I get and the more I don’t want to get up."*

Participant 681, woman from metropolitan centre in Ontario.

In contrast, cues reminding participants of their sedentary behaviour served as automatic motivation to facilitate reductions in prolonged sedentary behaviour. These included physical cues, such as feeling stiff and sore when engaging in prolonged sedentary behaviour, as well as external cues, such as notifications sent from technological devices or activity trackers. The utility of these prompts was largely related to participants’ reflective motivation, since they believed that reductions in sedentary time would be beneficial for both their physical and mental health.

*"When I don’t wear my watch, I might not get those reminders. So, what I do is I try to wear it all the time so that at least the beep will make me go ‘ok, you got to get up'."*

Participant 520, man from city in Alberta.

Perceived benefits of and personal goals related to reducing prolonged sedentary behaviour were endorsed by all participants, highlighting reflective motivation as an important facilitator across domains.

*I’m just sitting all day*,* and I know that it’s bad for my body. It’s just hard to not do it.”*

Participant 055, woman from city in Ontario.

### Recommendations for intervention development

Following the BCW stages of intervention development, our analysis identified 43 behaviour change techniques (BCTs) for reducing prolonged sedentary behaviour, with varying relevance across domains (Table 3). Details of links between the TDF, COM-B, BCW, and BCTTv1 are reported for each sedentary behaviour domain and behavioural determinant in Supplementary Material 3.

**Table 3:**
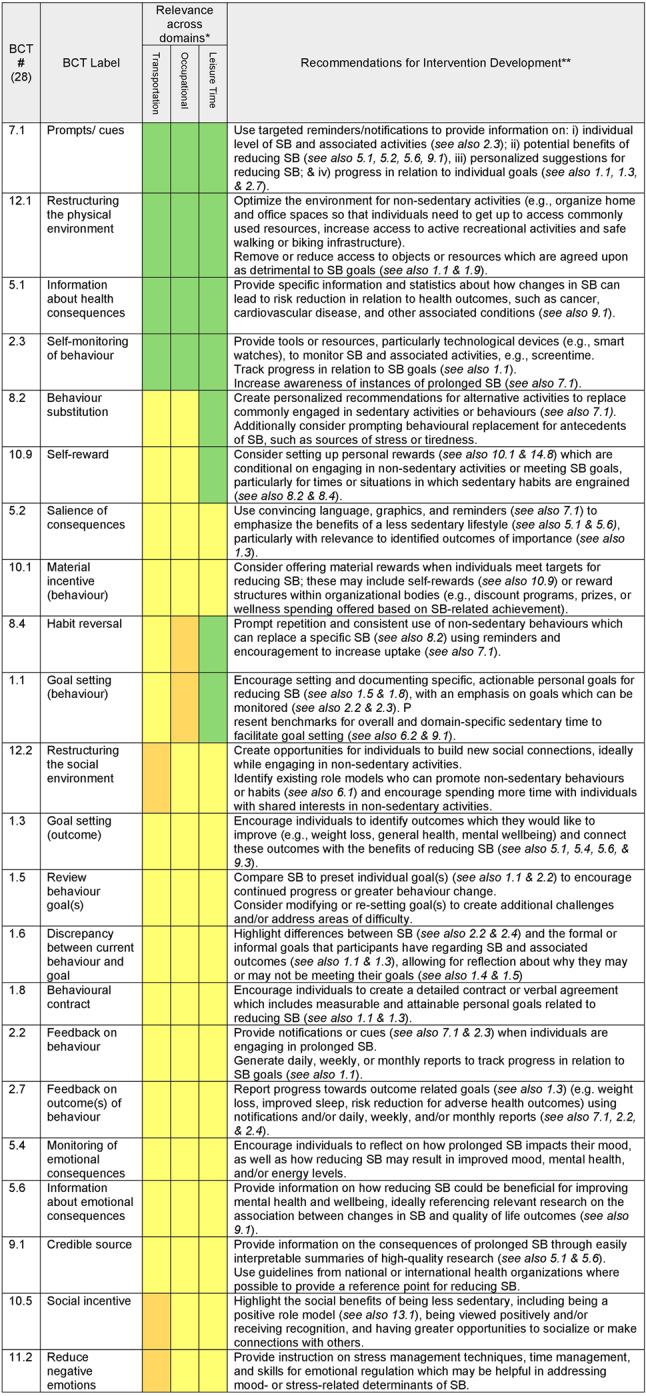

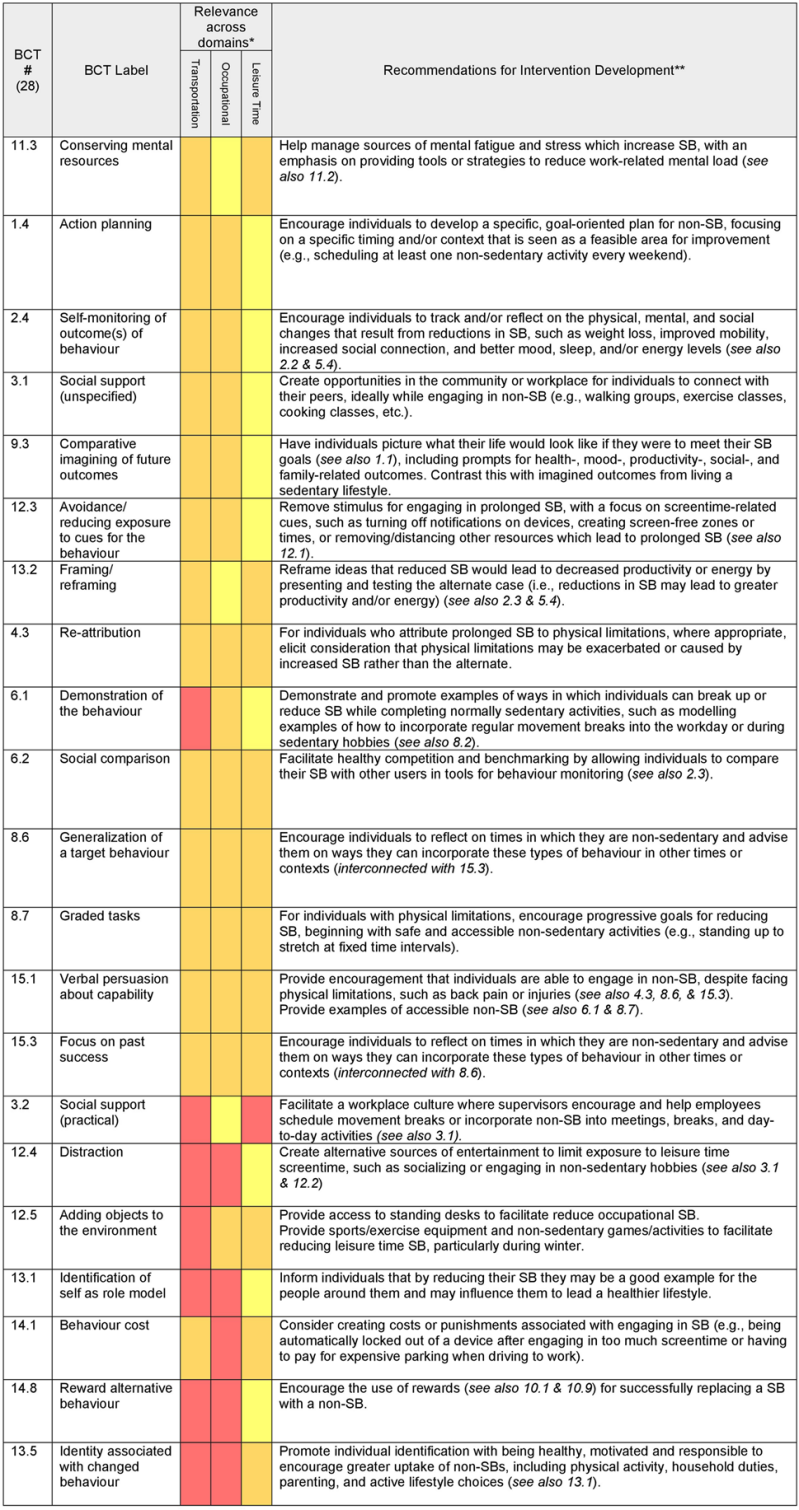
Behaviour Change Techniques (BCTs) identified from qualitative interviews, sorted by relevance to determinants of reducing prolonged sedentary behaviour (SB) and classified by SB domain

* Relevance across domains was assessed by determining the number of behavioural determinants which were associated with each technique based on analysis of the qualitative interviews (See Supplementary Material 3). Determinants of general sedentary behaviour were considered to be associated with every domain)

** For detailed determinant-specific examples, see descriptions of intervention functions outlined in Supplementary Material 3

The most commonly identified BCT was the use of prompts/cues, which were viewed as useful for leveraging facilitators related to belief in consequences and responsiveness to notifications, as well as helping to overcome barriers related to automatic motivation and behavioural regulation. These prompts/cues were frequently mentioned along with goal setting, feedback, and self-monitoring. The ideal delivery of prompts/cues was commonly described as incorporating information about individuals’ personal level of sedentary behaviour in relation to their personal goals for behaviour change. Furthermore, motivation-related determinants were commonly addressed by functions of incentivization in that participants viewed various forms of rewards or incentive structures as beneficial for encouraging reductions in prolonged sedentary behaviour.

Capability-related determinants, which spanned all domains of sedentary behaviour, were largely related to education functions, wherein information about health consequences and the importance of these consequences could be used to address gaps in knowledge. In relation to opportunity-related determinants, participants referenced techniques to restructure the physical and social environment and create a context that encourages reductions in sedentary behaviour. These techniques were viewed as important across domains of sedentary behaviour, however, wider policy-level interventions were often referred to for modifying transportation sedentary behaviour, in contrast to leisure time and occupational sedentary behaviour which were viewed as being more influenced by local changes, such as adding objects to the environment.

Considering interconnected links between capability, opportunity, motivation and behaviour, high-priority BCTs varied across domains of sedentary behaviour (Supplementary Material 4). Based on participant perspectives and mapped behavioural determinants, transportation sedentary behaviour was most frequently linked to policy-level strategies focused on restructuring the physical environment. In contrast, occupational sedentary behaviour was commonly linked to strategies targeting the workplace social environment to address barriers in social opportunity. While barriers related to automatic motivation were described by participants as broadly applicable across domains, they were particularly prominent within the leisure time domain, which was characterized by relatively few opportunity-related barriers. As such, a focus on individual-level strategies such as self-monitoring supported by goal-oriented prompts/cues were identified as especially relevant for reducing leisure time sedentary behaviour.

## Discussion

This study used the BCW to guide the systematic analysis of qualitative interviews and identify key components for developing domain-tailored intervention strategies to reduce prolonged sedentary behaviour among Canadian adults. Determinants of sedentary behaviour across domains were contingent on interrelated sources of behaviour from each category of the COM-B model. Most participants expressed an interest in reducing sedentary behaviour to improve their personal health and wellbeing, particularly highlighting leisure time sedentary behaviour as an area for improvement. As such, interventions aimed at reducing prolonged sedentary behaviour may be well aligned with participants’ reflective motivation. Nevertheless, their effectiveness is likely dependent on incorporating active ingredients that address domain-specific barriers related to automatic motivation, psychological capability, physical and social opportunity.

Consistent with the BCTs identified in our qualitative investigation, a review of behaviour change interventions also highlighted self-monitoring and restructuring the social or physical environment as promising techniques to reduce sedentary behaviour among adults [29]. Our study extended these findings by conducting behavioural context-specific investigations to target and tailor interventions to transportation, occupational and leisure time domains of sedentary behaviour. While prior studies have applied a behavioural approach to intervention development targeting a single domain of sedentary behaviour, for example in occupational settings [30–33], to the best of our knowledge our study is the first to investigate distinct determinants of sedentary behaviour across domains.

By investigating domain-specific determinants of sedentary behaviour, our study provides unique insights on how prominent sources of behaviour, and thereby corresponding intervention strategies, may vary depending on the context of the behaviour. Key barriers identified in the transportation domain centred on physical opportunity, the occupational domain centred on social opportunity, and the leisure time domain centred on automatic motivation. These findings can be considered from an ecological perspective, which posits that behaviour both affects and is affected by multiple levels of influence [34]. Applying this perspective to consider differences across domains, participant accounts suggested that policy-level intervention may be most relevant for reducing transportation sedentary behaviour, organizational-level intervention for occupational sedentary behaviour, and individual-level intervention for leisure time sedentary behaviour. Furthermore, the multilevel intervention development strategies identified in our analysis are consistent with a whole-system approach, as reflected in the World Health Organization’s Global Action Plan on Physical Activity 2018–2030, which combines “upstream” policy and “downstream” individual interventions to achieve a coordinated system-level response to reverse sedentary trends [35].

Findings from our qualitative analysis suggest that future health promotion efforts aimed at reducing sedentary behaviour may benefit from considering different levels of influence across transportation, occupational and leisure time contexts. Interventions at the individual-level, such as personal applications or devices to increase motivation, while reasonably useful across domains, fail to address deficits in opportunity, such as the surrounding built environment and cultural context. In contexts with more prominent opportunity-related barriers, organizational- and policy-level efforts, such as implementing workplace policies to encourage movement or funding projects to improve community walkability and bike-ability may be needed to enable changes in behaviour. Furthermore, these various levels of influence are interdependent, and individual-level interventions should assess capability- and opportunity-related barriers to offer recommendations of enhanced personal relevance, tailored to interrelated factors determining sedentary behaviour.

Despite differences across domains, many determinants of sedentary behaviour were viewed by participants as broadly applicable and may be meaningful targets for interventions to reduce overall sedentary behaviour. Significantly, our study identified psychological capability barriers that applied across domains, as participants lacked knowledge regarding the appropriate or target amount of sedentary behaviour. Current WHO guidelines recommend limiting sedentary behaviour, but no specific time range or cut-off is recommended [9, 10]. In contrast, targets for limiting daily sedentary behaviour are available within Canadian 24-hour movement guidelines, however, participants were generally unaware of this recommendation. The difference between Canadian and international guidelines highlights a lack of consensus on quantified recommendations for sedentary behaviour, as less evidence is available compared with physical activity. To allow for better goal setting and increased motivation for reducing sedentary behaviour, more research is needed to provide information on patterns of sedentary behaviour which are associated with significant health benefits.

### Strengths and limitations

By using a theory-informed, systematic analysis of qualitative data, our findings are useful for informing targeted interventions to reduce domain-specific sedentary behaviour among Canadian adults. Although use of the BCW allowed for translating qualitative findings into practical intervention components, its theory-informed structure may have constrained thematic interpretation and limited openness to emergent or nuanced insights during analysis. Additionally, interviews varied in length and participant engagement. Although analytic saturation was achieved for domain-specific determinants, shorter interviews may have limited the exploration of less frequently discussed barriers in certain domains.

The trustworthiness of our findings is supported by the use of methods to support the study’s credibility and dependability, including data triangulation, reflexivity, and detailed documentation of the research process throughout data collection and analysis. Additionally, purposive sampling was used to recruit a sample of participants with diverse sociodemographic background across age, gender, race, and residency status in Canada. Nevertheless, while there were a large number of respondents to our screening questionnaire, we were unable to recruit any participants from Canada’s northern territories, limiting the transferability of our findings to these regions. Furthermore, the study sample included a higher proportion of younger participants and participants with white racial/ethnic identities, which may limit transferability to populations with distinct sociodemographic characteristics. Additionally, several important behavioural determinants were specific to the geography and built environment in Canada, referencing winter climate and significant distances between municipalities or amenities; hence, the determinants of sedentary behaviour may vary significantly for individuals outside of the Canadian context.

## Conclusions

This study outlines a theory-informed, end-user-engaged intervention development process which aims to reduce prolonged sedentary behaviour among Canadian adults. Through analyzing domain-specific determinants of reducing prolonged sedentary behaviour, an intervention strategy which incorporates behaviour change techniques involving goal-oriented prompts/cues based on monitoring of sedentary behaviour was identified. Additionally, restructuring the social and physical environment to enable and encourage non-sedentary behaviour is recommended. Although individual-level interventions aimed at increasing motivation may be useful for reducing prolonged sedentary behaviour, broader organizational and policy-level changes may be required, particularly for targeting occupational and transportation sedentary behaviour.

## Supplementary Information

Supplementary Material 1.

Supplementary Material 2.

Supplementary Material 3.

Supplementary Material 4

## Data Availability

The datasets generated and analyzed during the current study are not publicly available due to participant privacy and confidentiality but may be made available from the corresponding author on reasonable request.

## Abbreviations

BCT: Behaviour Change Technique
BCTTv1: Behaviour Change Technique Taxonomy, version 1
BCW: Behaviour Change Wheel
COM-B: Capability, opportunity, motivation – behavior
TDF: Theoretical Domains Framework
WHO: World Health Organization
