# Supplementary material for: Intervention development to reduce sedentary behaviour among adults: a qualitative investigation using the Behaviour Change Wheel
Source: Int J Behav Nutr Phys Act. 2026 Apr 21;23:62. doi: 10.1186/s12966-026-01917-w (PMC13255465; doi:10.1186/s12966-026-01917-w)
Supplement: Supplementary file 1 — Supplementary Material 1. [file 12966_2026_1917_MOESM1_ESM.docx]

| **Appendix 1: Recruitment framework for purposive sampling of interview participants to ensure diversity in sociodemographic characteristics represented within the Canadian population, developed in collaboration with the Canadian Cancer Society** | | | | | |
| --- | --- | --- | --- | --- | --- |
| **Equity Factor** | **Recruitment Goal** | **Responded (n=593)** | **Contacted (n=57)** | **Interviewed (n=30)** | **Goal Met** |
| Geography | At least 3 representatives from British Columbia | 108 | 6 | 5 | Y |
|  | At least 5 representatives from the Prairie region (AB, SK, MB) | 133 | 17 | 10 | Y |
|  | At least 3 representatives from Ontario | 243 | 19 | 9 | Y |
|  | At least 3 representatives from Quebec | 70 | 5 | 2 | N  (-1) |
|  | At least 5 representatives from the Atlantic region (PEI, NFL, NB, NS) | 38 | 9 | 4 | N  (-1) |
|  | At least 1 representative from the territories (NU, YK, NWT) | 1 | 1 | 0 | N  (-1) |
| Rurality | At least 12 representatives from rural and small towns | 230 | 23 | 11 | N  (-1) |
| Age | At least 8 representatives aged 25 to 44 years old | 252 | 30 | 18 | Y |
|  | At least 8 representatives aged 45 to 64 years old | 228 | 18 | 9 | Y |
|  | At least 8 representatives aged 65 years old and over | 112 | 9 | 3 | N  (-5) |
| Racial or ethnic background | At least 18 representatives from racialized communities | 179 | 30 | 15 | N  (-3) |
|  | At least 3 representatives that identify as Indigenous (First Nations, Inuit or Métis) | 23 | 6 | 2 | N  (-1) |
|  | At least 3 representatives that identify as Black | 27 | 4 | 2 | N  (-1) |
| Gender identity | At least 10 representatives that identify as a man | 118 | 16 | 10 | Y |
|  | At least 10 representatives that identify as a woman | 469 | 39 | 19 | Y |
|  | At least 3 representatives that identify as non-binary or third gender or two-spirit or other (self-describe) | 3 | 1 | 1 | N  (-2) |
|  | At least 1 representative that identifies as transgender | 2 | 1 | 1 | Y |
| Sexual orientation | At least 5 representatives that identify as gay or lesbian, bisexual, or other (self-describe) | 49 | 10 | 8 | Y |
| Income | At least 1 representative with an income between $0 and $39,999 | 122 | 14 | 6 | Y |
|  | At least 2 representatives with an income between $40,000 and $79,999 | 217 | 17 | 12 | Y |
|  | At least 2 representatives with an income between $80,000 and $119,999 | 125 | 18 | 8 | Y |
|  | At least 1 representative with an income above $120,000 | 56 | 4 | 1 | Y |
| Residency status | At least 1 representative that is a new immigrant | 8 | 1 | 0 | N  (-1) |
|  | At least 1 representative that is a permanent resident | 44 | 6 | 3 | Y |

Following interviews with 30 participants, Canadian Cancer Society partners reviewed recruitment targets in comparison to characteristics within the study sample; CCS stated that they found the study sample to be sufficiently representative of sociodemographic characteristics within the Canadian population and recruitment was ended.
