# Supplementary material for: Intervention development to reduce sedentary behaviour among adults: a qualitative investigation using the Behaviour Change Wheel
Source: Int J Behav Nutr Phys Act. 2026 Apr 21;23:62. doi: 10.1186/s12966-026-01917-w (PMC13255465; doi:10.1186/s12966-026-01917-w)
Supplement: Supplementary file 2 — Supplementary Material 2. [file 12966_2026_1917_MOESM2_ESM.docx]

**Appendix 2: Semi-structured interview guide for exploring participants’ barriers and facilitators to reducing prolonged sedentary behaviour**

| **COM-B** | **TDF domain** | **Eliciting questions** |
| --- | --- | --- |
| Capability  Psychological | Knowledge | Can you start by telling me your understanding of current advice by experts about how much sitting time is okay?  Prompt—At what point do you think sitting becomes too much?  Prompt—Do you know of any consequences from sitting for long periods?  How do you think your sitting time compares with this advice? |
|  | Memory, attention and decision processes | How do you decide when to take a break from sitting or to get up and move?  Do you find it easy or difficult to remember to limit your sitting time while managing other responsibilities?  What do you think could help you overcome barriers that might prevent you from breaking up and reducing your sitting [at work, at home, during social activities, for transport]? |
|  | Behavioural regulation | Do you monitor or keep track of how long you sit or how often you take breaks?  What would need to change to help you break up long periods of sitting at [at work, at home, during social activities, for transport]? |
| Capability  Physical | Skills | Do you have any physical limitations that prevent you from breaking up or reducing your sedentary time?  Are there any other things that might prevent you from breaking up and reducing your sitting time [at work, at home, during social activities, for transport]? |
| Opportunity  Social | Social influences | How do the people you [work, live, socialize, travel] with influence your sitting time?  How could your [colleagues, employer, family members, friends] help you to break up and reduce your sitting time? |
| Opportunity  Physical | Environmental context and resources | How does your environment [at work, at home, for social activities, for transport] influence your sitting behaviour? If you [lived, worked] in a different place, do you think your sitting time would be different? Why?  How would your environment need to change to make breaking up and reducing sitting easier for you? |
| Motivation  Reflective | Beliefs about capabilities | How confident are you that you could break up your sitting time [at work, at home, during social activities, for transport]? |
|  | Social/professional roles and identify | Do you feel that your role [at work, at home] influences whether you can take breaks to move around?  If you were in a different role, do you think your daily sitting patterns would look different? Why or why not? |
|  | Beliefs about consequences | How much benefit do you feel breaking up your sitting time would give you? |
|  | Intentions, goals | Do you want to reduce the amount of time you spend being sedentary?  Have you ever set goals to reduce how much you sit or to move more during the day?  In what area do you think reducing your sitting time would be the most beneficial [at work, at home, during social activities, for transport]? |
| Motivation  Automatic | Emotion | How do you think your mood during the day would influence your sitting patterns? |
|  | Reinforcement | How do you think your habits or routines influence your sitting patterns?  How could you overcome these to break up and reduce your sitting time [at work, at home, during social activities, for transport]?  Are there any benefits or incentives that make it easier or harder for you to reduce the amount of time you spend sitting? |
