# Supplementary material for: Intervention development to reduce sedentary behaviour among adults: a qualitative investigation using the Behaviour Change Wheel
Source: Int J Behav Nutr Phys Act. 2026 Apr 21;23:62. doi: 10.1186/s12966-026-01917-w (PMC13255465; doi:10.1186/s12966-026-01917-w)
Supplement: Supplementary file 3 — Supplementary Material 3. [file 12966_2026_1917_MOESM3_ESM.docx]

**Appendix 3: Behavioural determinants of reducing sedentary behaviour (SB) with links between COM-B model, TDF domains, intervention functions, policy categories and behaviour change techniques (BCTs) for:**

[General sedentary behaviour (Table A3.1) pgs 2](#_Toc205563946)-7

[Transportation sedentary behaviour (Table A3.2) pgs 8](#_Toc205563947)-9

[Occupation sedentary behaviour (Table A3.3) pgs 10](#_Toc205563948)-12

[Leisure time sedentary behaviour (Table A3.4) pgs 13](#_Toc205563949)-18

**Table A3.1: Behavioural determinants of reducing general sedentary behaviour (SB) with links between COM-B model, TDF domains, intervention functions, policy categories and behaviour change techniques (BCTs)**

| COM-B | TDF | Determinant of Behaviour | Evidence from Interviews | Intervention Functions with associated recommendations | Policy categories | BCTs |
| --- | --- | --- | --- | --- | --- | --- |
| Psychological Capability | Knowledge | Lack of specific knowledge regarding SB guidelines  (barrier) | “I haven’t heard about sitting time. Just everybody says you need to get some exercise.” Participant 451  “I really haven't heard anything. This is actually the first study I've ever heard of being conducted… Based on practicality, I would think that you probably shouldn't be sitting for longer than- I don't know… I wouldn't even know what to guess.” Participant 555 | ***Education:*** Increase awareness of existing guidelines to provide a reference point for reducing SB and improve understanding of the potential benefits. | Guidelines; Communication/Marketing | 5.1 Information about health consequences  1.1 Goal setting (behaviour)  9.1 Credible source |
|  |  | Awareness that sedentary lifestyle can lead to negative health impacts (facilitator) | “Basically, all your health is jeopardized if you sit more- if you have a sedentary lifestyle.” Participant 492  “Well, the sedentary lifestyle does lead into obesity and all that stuff, so that I can sort of guess the consequences of that and the hypertension and all that spiel- like cardiovascular risks.” Participant 681 | ***Persuasion:*** Use convincing language and graphics to emphasize the benefits of a less sedentary lifestyle in order to encourage greater improvements in breaking up or reducing SB. | Communication/Marketing | 1.3 Goal setting (outcome)  5.1 Information about health consequences  5.2 Salience of consequences  9.3 Comparative imagining of future outcomes |
|  |  | Lack of specific knowledge on health benefits (barrier) | “I think this goes to the ‘What's the big benefit of it?’ So again, there's these abstract notions of like it's better for your health, right? But if you had a more of a metric [that would help me]” Participant 656 | ***Education:*** Provide specific information and statistics about how changes in SB can lead to risk reduction in relation to health outcomes, such as cancer. | Communication/Marketing | 5.1 Information about health consequences  5.2 Salience of consequences  7.1 Prompts/cues  9.1 Credible source |
|  | Behavioural Regulation | Difficulties making changes or breaking existing habits (barrier) | “My own mindset works against me because I've lately found myself to be like ‘oh, I'm so lazy, oh, I need to work out more’...so that would be the only barrier is my excuses.” Participant 679 | ***Enablement:*** Provide tools (mobile applications, activity trackers, watches, etc.) to help individuals set actionable SB goals, track progress, and provide reminders to promote change in behaviour. | Environmental/ social planning | 1.4 Action planning  2.3 Self-monitoring of behaviour  7.1 Prompts/cues  8.2 Behaviour substitution  8.4 Habit reversal |
| Physical Capability | Physical Skills | Limitations or injuries may make it difficult to break up SB (barrier) | “I’ve got back problems that also inhibit my mobility at times, so that doesn't help.” Participant 683 | ***Training:*** Provide direction on ways to engage in safe and accessible non-sedentary activities for individuals with physical limitations.  ***Education:*** Provide information on how reducing SB could be beneficial for improving back pain and other relevant physical health concerns. | Service provision | 8.7 Graded tasks  15.1 Verbal persuasion about capability  4.3 Re-attribution |
|  |  | Physically able to engage in non-sedentary activities (facilitator) | “It's a total of almost an hour of walking there and back and it's no big deal.” Participant 691 | ***Modelling:*** Provide examples of ways that individuals in similar situations engage in non-sedentary activities throughout their daily routine. | Communication/Marketing | 8.6 Generalization of a target behaviour  15.3 Focus on past success |
| Social Opportunity | Social Influences | Interacting with friends, family, and co-workers (facilitator) | “I could easily go for a walk, but it's just I would be walking by myself. It's less- there's less onus on me to do it because it would be out of my own self-discipline as opposed to just being put on you and as a social thing” Participant 233  “I think my family helps me spend less time on my screen… if I was alone, I'd probably just be on my phone.” Participant 704 | ***Environmental restructuring:*** Create opportunities in the community or workplace for individuals to connect with their peers, ideally while engaging in non-sedentary behaviours (e.g., walking groups, exercise classes, cooking classes, etc.).  ***Modelling:*** Encourage using social connections who engage in non-sedentary behaviours as positive examples to emulate. | Environmental/ social planning; Service Provision | 3.1 Social support (unspecified)  12.2 Restructuring the social environment  10.5 Social incentive |
| Physical Opportunity | Environmental Context and Resources | Poor weather and shorter days in the wintertime (barrier) | “During wintertime, it's awful for me, I'm going to be lazy and I'm going to be like, ‘I don't want to go outside.’ I don't have any urge to go outside” Participant 628  “As the weather starts to get nicer, I think that'll help. Again, too that it's easier to not feel like it's bedtime at 8:00 o'clock because it's already pitch black out...Yeah, I think a lot of mine seems to be seasonal.” Participant 660 | ***Enablement:*** Increase access to indoor recreational activities, for example using vouchers or discounts for membership to indoor non-sedentary activities, like gyms, recreation centres, pools, or activity venues, such as curling or bowling.  ***Training:*** Teach skills related to winter sports (such as skiing, snowshoeing, or skating) or non-sedentary indoor activities which can be done year-round. | Environmental/ social planning; Service Provision | 12.1 Restructuring the physical environment  8.2 Behavioural substitution |
|  |  | Creating physical distance between self and objects (facilitator) | “I just try to keep my phone or the mobile phone at a distance because that compels me to move all the way. And I scatter few [things] over my workplace, I just don't keep everything in my vicinity. I just ensure that it's somewhere at least a meter or like 3 feet so that I can just go and take it” Participant 171 | ***Environmental restructuring:*** Create designated spots for commonly used items, such as phone, snacks, or drinks, which require getting up from a seated position to access them. This may also include work-related resources, such as calendars, pens, or whiteboards. | Environmental/ social planning | 12.1 Restructuring the physical environment |
| Automatic Motivation | Emotion | Cyclical relationship between poor mood and increased SB (barrier) | “It's like a negative feedback loop. The more I sit down, the more grumpy I get and the more I don't want to get up” Participant 681 | ***Education:*** Provide information on how reducing SB could be beneficial for improving mental health, energy levels, and wellbeing.  ***Training:*** Provide instruction on stress management techniques and skills for emotional regulation. | Communication/Marketing; Service Provision | 5.4 Monitoring of emotional consequences  5.6 Information about emotional consequences  11.2 Reduce negative emotions |
|  |  | Feeling drained or tired (barrier) | “It depends on how much I have on my plate that week and how much brain power I use… if I only have the capacity to just hang out and watch TV with a friend then that's kind of what I'll do. But it really depends on the capacity I have after that day” Participant 713  “I could exercise more, but that means I have to wake up earlier and I'm already tired.” Participant 684 | ***Education:*** Provide information on how reducing SB could be beneficial for improving mental health, energy levels, and wellbeing.  ***Training:*** Provide instruction on stress management techniques, sleep hygiene, and time management to help manage or reduce feelings of tiredness. | Communication/Marketing; Service Provision | 11.3 Conserving mental resources  13.2 Framing/ reframing  5.4 Monitoring of emotional consequences  5.6 Information about emotional consequences |
|  | Reinforcement | Reminders to break up sitting time (facilitator) | “When I don't wear my watch, I might not get those reminders. So, what I do is I try to wear it all the time so that at least the beep will make me go ‘ok, you got to get up’” Participant 520 | ***Enablement:*** Provide tools (mobile applications, activity trackers, watches, etc.) to help individuals set actionable SB goals, track progress, and provide reminders to promote change in behaviour.  ***Incentivization:*** Consider offering material rewards, such as wellness spending allowances or prizes for individuals who meet movement goals/guidelines in order to increase responsiveness to prompts. | Fiscal | 7.1 Prompts/cues  2.2 Feedback on behaviour  2.3 Self-monitoring of behaviour  10.9 Self-reward  6.2 Social comparison  2.7 Feedback on outcome(s) of behaviour  10.1 Material incentive (behaviour) |
|  |  | Extended sitting time causes stiffness or soreness (facilitator) | “There are times where my back gets painful, and it helps to get up and move a little bit. But unless I've got that pain to prompt me, I tend to stay parked.” Participant 297 | ***Education:*** Provide information on how reducing SB could be beneficial for improving back pain and other relevant physical health concerns. | Communication/Marketing | 2.4 Self-monitoring of outcome(s) of behaviour  5.1 Information about health consequences |
| Reflective Motivation | Intentions & Goals | Desire to reduce amount of sedentary time (facilitator) | “I actually do get that thought in my head going: ‘You should get up.’” Participant 555  “I sit much more than I would like to. So, I try to get aware of it.” Participant 659 | ***Persuasion:*** Create a contract or verbal agreement with measurable and attainable personal goals related to reducing SB.  ***Incentivization:*** Encourage the use of self-administered rewards for adhering to set goals. For example, only buying coffee if you break up sitting time at least 5 times throughout the workday. | Communication/Marketing; Fiscal | 1.5 Review behaviour goal(s)  1.6 Discrepancy between current behaviour and goal  2.2 Feedback on behaviour  2.3 Self-monitoring of behaviour  2.7 Feedback on outcome(s) of behaviour  1.8 Behavioural contract  10.9 Self-reward  10.1 Material reward |
|  |  | Desire to reduce screentime (facilitator) | “Probably I am most interested in reducing screentime, but that's probably the most difficult one to also reduce.” Participant 704  “I could do with reducing screen time, that's for sure.” Participant 686 | ***Restriction:*** Remove or reduce access to technology (e.g., television, computers, tablets), websites, or platforms which are agreed upon as unnecessary and undesirable or detrimental.  ***Persuasion:*** Use cues on technological devices to monitor screentime and send reminders about the importance of limiting screentime and replacing it with non-sedentary behaviours. | Guidelines | 1.5 Review behaviour goal(s)  1.6 Discrepancy between current behaviour and goal  2.3 Self-monitoring of behaviour  12.1 Restructuring the physical environment  12.3 Avoidance/ reducing exposure to cues for the behaviour  1.8 Behavioural contract |
|  | Belief in Consequences | General belief that being more active and/or less sedentary would benefit short-term and long-term health (facilitator) | “It's actually not healthy to sit. As a human, we are not supposed to be sitting almost at all because it's not natural to our bodies, so even standing is better than sitting. So, I know that it's actually really, really bad for you.” Participant 679 | ***Persuasion:*** Use targeted messaging and reminders to emphasize the positive impact that breaking up or reducing SB can have on health. Reference and present guidelines for daily SB to encourage setting a personal goal for amount of sedentary time per day. | Communication/Marketing; Guidelines | 1.3 Goal setting (outcome)  5.2 Salience of consequences  7.1 Prompts/cues |

**Table A3.2: Behavioural determinants of reducing transportation sedentary behaviour (SB) with links between COM-B model, TDF domains, intervention functions, policy categories, and behaviour change techniques (BCTs)**

| COM-B | TDF | Determinant of Behaviour | Evidence from Interviews | Description of intervention functions | Policy categories | BCTs |
| --- | --- | --- | --- | --- | --- | --- |
| Physical Opportunity | Environmental Context and Resources | Significant distance between home and common destinations (amenities or work) (barrier) | “If I lived closer, I would absolutely walk, but when it's a 45 minute to hour long walk, it makes it tough to do that.” Participant 673  “I live in a small community, there's only a convenience store here, so I wouldn't do my grocery shopping there. I have to go to the next town” Participant 053 | ***Environmental restructuring:*** Improve urban & rural planning to increase access to amenities. | Environmental/ social planning; Fiscal | 12.1 Restructuring the physical environment |
|  |  | Limited access to public transportation, particularly in smaller communities (barrier) | “So, I live in a really rural area... there's no transit system, there's no bike lanes, there's none of that. So, your options are basically walking, which you can't do because everything is so far apart. So, you need to drive.” Participant 660 | ***Environmental restructuring:*** Improve urban & rural planning to increase access to public transportation. | Environmental/ social planning; Fiscal | 12.1 Restructuring the physical environment |
|  |  | Lack of accessible and safe walking or bike paths (barrier) | “There are bike lanes, but again they are also not properly maintained by the town. There's not a lot of nature trails in my area. You have to drive out of this area to get to them.” Participant 555 | ***Environmental restructuring:*** Improve urban & rural planning to increase access to walking and biking infrastructure. | Environmental/ social planning | 12.1 Restructuring the physical environment |
| Automatic Motivation | Reinforcement | Ingrained habits regarding driving as primary transport (barrier) | “Things are kind of in walking distance, but I tend to drive...It's just a mentality of not walking. If I wanted to, there's absolutely nothing to stop it.” Participant 451 | ***Persuasion:*** Use tools for monitoring and feedback to send targeted reminders about choice of transportation in relation to set goals/guidelines  ***Coercion:*** Consider using parking costs as a deterrent, for example creating free storage spaces for employee bikes, while motor vehicle parking lots have a charge.  ***Incentivization:*** Consider creating reward structures for choosing active transportation, for example discount programs for car insurance based on driving mileage. | Communication/Marketing; Fiscal | 8.2 Behaviour substitution  8.4 Habit reversal  1.1 Goal setting (behaviour)  14.1 Behaviour cost  10.1 Material incentive (behaviour)  7.1 Prompts/cues  2.3 Self-monitoring of behaviour |

**Table A3.3: Behavioural determinants of reducing occupation sedentary behaviour (SB) with links between COM-B model, TDF domains, intervention functions, policy categories, and behaviour change techniques (BCTs)**

| COM-B | TDF | Determinant of Behaviour | Evidence from Interviews | Description of intervention functions | Policy categories | BCTs |
| --- | --- | --- | --- | --- | --- | --- |
| Social Opportunity | Social Influences | Workplace culture that encourages breaks (facilitator) | “It is really encouraged, and our manager constantly reminds us to take care of our overall wellness, to have movement breaks throughout the day” Participant 233 | ***Enablement:*** Reduce social barriers by facilitating opportunities to get to know co-workers and increase familiarity and comfort within the workplace.  ***Environmental restructuring:*** Consider creating areas in the workplace that encourage non-sedentary breaks, such as using a standing height table/desk to set up puzzles or other activities.  ***Persuasion:*** Encourage and set aside time to take short movement breaks during the workday; promote these priorities in newsletters, scheduling tools, or other workplace communications. | Environmental/ social planning; Regulation | 10.5 Social incentive  12.2 Restructuring the social environment  10.1 Material incentive (behaviour)  3.2 Social support (practical)  6.1 Demonstration of the behaviour |
| Physical Opportunity | Environmental Context and Resources | Workplace resources to help decrease SB, such as a standing desk (facilitator) | “I did used to have a standing desk... I loved it. I notice substantial differences if I compare then and now” Participant 233 | ***Environmental restructuring:*** Provide resources, such as standing desks & walking pads that enable work to be done while standing or moving.  ***Enablement:*** For hybrid or remote workers, consider providing reimbursement for employees who purchase resources to facilitate non-sedentary behaviour in their home workspace. | Environmental/ social planning; Fiscal; Regulation | 12.5 Adding objects to the environment |
|  |  | Working from home allows for increased flexibility and autonomy * (facilitator) | “I have a lot of flexibility that I wouldn't get in other jobs… it's a little easier when I'm at home to maybe [take breaks], go empty the dishwasher or whatever, throw a load of laundry in.” Participant 684 | ***Environmental restructuring:*** Consider creating areas in the workplace that encourage non-sedentary breaks, such as using a standing height table/desk to set up puzzles or other activities. | Regulation | 12.1 Restructuring the physical environment  12.2 Restructuring the social environment |
| Reflective Motivation | Professional Role/ Identity | Challenges keeping up with expectations at work (barrier) | “I feel like if I sit in my desk all day and get my work done, then I don't have to work late…the more I stand up and go talk the less I'm going to get done” Participant 374  “When it's a busy day, I didn't even notice I am sit[ting] all the time because I am doing a lot of things.” Participant 579 | ***Training:*** Undertake training to improve time management skills and build workplace schedules or plans which include breaks in SB.  ***Modelling:*** Demonstrate and promote examples of ways in which employees can productively break up or reduce SB while completing work-related tasks. | Regulation; Communication/ Marketing | 1.2 Problem solving  11.2 Reduce negative emotions  11.3 Conserving mental resources |
|  |  | Need to focus for extended periods or ‘get in the zone’ to accomplish occupational tasks (barrier) | “[My work] requires getting into things. So, it's very difficult to just do something for half an hour… So, I have a tendency to not want to break things up because I lose my train of thought.” Participant 492 | ***Persuasion:*** Use reframing to emphasize the potential that breaking up SB could actually lead to greater productivity and/or creativity by allowing for mental breaks and a fresh perspective on tasks. Use reminders to encourage active mental breaks. | Communication/ Marketing | 13.2 Framing/ reframing  7.1 Prompts/ cues  3.2 Social support (practical) |

* This facilitator should be considered in conjunction with the General SB facilitator *Interacting with friends, family, and co-workers,* which was generally viewed as less influential when working from home, compared to an in-person setting

**Table A3.4: Behavioural determinants of reducing leisure time sedentary behaviour (SB) with links between COM-B model, TDF domains, intervention functions, policy categories, and behaviour change techniques (BCTs)**

| COM-B | TDF | Determinant of Behaviour | Evidence from Interviews | Description of intervention functions | Policy categories | BCTs |
| --- | --- | --- | --- | --- | --- | --- |
| Social Opportunity | Social Influences | Shared interests with friends/family in TV, video games, or virtual activities (barrier) | “I've thought about [getting rid of my TV] but it's usually the other people around me who are like, ‘Are you sure you want to do that?’ or my boyfriend will be like, ‘Well, then what are we going to watch wrestling on?’ And I'm like, well, I love wrestling, but I would rather be going for walks, to be honest.” Participant 008 | ***Persuasion:*** Encourage individuals to view themselves as positive role models for reducing screentime and SB within their social groups.  ***Enablement:*** Increase access to non-sedentary social activities, for example hosting free or low-cost social events, like bowling, skating, or craft nights (at standing height tables). | Communication/ Marketing; Environmental/ social planning | 13.1 Identification of self as role model  12.2 Restructuring the social environment |
|  |  | Social isolation (barrier) | “When I'm at home, I'm sitting most of the time… I don't have anybody right now that I- Other than my dog- there's nothing else.” Participant 045  “When I used to live at home I would have a parent come in and be like, ‘you haven't left your room all day, you should move around and do something.’ But now that I don't, I can just stay in my room all day for days on end and no one will say anything.” Participant 055 | ***Environmental restructuring:*** Create opportunities in the community for individuals to connect with their peers, ideally while engaging in non-sedentary behaviours (e.g., walking groups, exercise classes, cooking classes, etc.). | Environmental/ social planning | 3.1 Social support (unspecified)  12.2 Restructuring the social environment |
| Physical Opportunity | Environmental Context and Resources | Widespread access to technological devices for entertainment/ screentime  (barrier) | “I think what would be a really big help is not having any screen in my room or in my bed with me” Participant 673  “I don't watch very much TV now, but I do go on my phone.” Participant 684 | ***Environmental restructuring:*** Create designated spots for devices, such as phone, tablets, or gaming consoles, which requires effort to access them or limits their use, e.g., keep phone in a timed lock box that can’t be opened for a certain amount of time.  ***Restriction:*** Remove or reduce access to technology (e.g., television, computers, tablets), websites, or platforms which are agreed upon as unnecessary and undesirable or detrimental. For example, deleting social media accounts, blocking websites or creating limits for TV/screentime. | Guidelines; Regulation | 12.1 Restructuring the physical environment  12.3 Avoidance/ reducing exposure to cues for the behaviour  12.4 Distraction |
| Automatic Motivation | Reinforcement | Routine involves ‘winding down’ in the evenings (barrier) | “I finish eating, put away my food, and then I go and lounge because that's just what I do.” Participant 673 | ***Persuasion:*** Use tools for monitoring and feedback to send targeted reminders which include personalized recommendations for alternative activities to break up SB in the evenings.  ***Incentivization:*** Consider using rewards to encourage non-sedentary activities after dinner, for example walking to a shop to get dessert. | Communication/ Marketing; Fiscal | 8.2 Behaviour substitution  8.4 Habit reversal  14.8 Reward alternative behaviour  10.1 Material incentive (behaviour)  10.9 Self-reward |
|  |  | Screentime activities are addicting and easy to get lost in (barrier) | “I get sucked in to a show and keep wanting to watch the next episode. Sometimes there will be times and I'm like ‘I really need to use the washroom, but this show is too good’ and I just won't even get up to do that.” Participant 173  “I just kind of get sucked into my phone. Like for social media, I kind of get sucked in and then- yeah, but there's nothing really preventing me from not going on social media, I could just not, but I don't make that choice.” Participant 704 | ***Restriction:*** Remove or reduce access to technology (e.g., television, computers, tablets), websites, or platforms which are agreed upon as unnecessary and undesirable or detrimental.  ***Incentivization:*** Consider using rewards when goals regarding screentime are met (e.g., buying yourself a reward if weekly screentime is <7 hours) or if goals regarding non-screentime activities are met (e.g., get ice cream when going for a walk after dinner instead of watching TV). | Regulation; Communication/ Marketing | 1.1, Goal setting (behaviour)  7.1 Prompts/cues  12.4 Distraction  14.1 Behaviour cost  14.8 Reward alternative behaviour  10.9 Self-reward |
|  |  | Sedentary activities are relaxing and can be a form of escape (barrier) | “To reduce the stress sometimes we sit to watch movies” Participant 463  “I actually prefer to watch things that are lighthearted and escapism to free up in my brain” Participant 492 | ***Training:*** Provide instruction on strategies for stress management which can replace screentime or other forms of sedentary escapism. For example, teaching mind-body exercises such as yoga or Tai Chi  ***Environmental restructuring:*** Consider modifying the home environment to encourage standing rather than sitting/lounging, for example adding a standing desk or removing very comfortable furniture which promotes prolonged SB. | Service provision; Environmental/ social planning | 8.2 Behaviour substitution  11.2 Reduce negative emotions  1.1, Goal setting (behaviour)  8.4 Habit reversal |
|  |  | Sedentary activities can be enjoyable by offering mental engagement or stimulation (barrier) | “It's a very comfortable sort of brain space for me where the game is different every time, so it's a little bit challenging. It involves some thinking and some creativity, but not so challenging that it doesn't feel like I'm working at it.” Participant 297 | ***Modelling:*** Demonstrate and promote examples of ways in which individuals can break up or reduce SB while completing mentally engaging activities, such as incorporating movement breaks into activities such as games or reading. | Communication/ Marketing | 8.2 Behaviour substitution  8.4 Habit reversal  6.1 Demonstration of the behaviour  12.5 Adding objects to the environment  1.1 Goal setting (behaviour) |
|  |  | Participating in active, non-sedentary hobbies can be rewarding and feel good (facilitator) | “I play ping pong or tennis table tennis, I ride motorcycle, I go hiking quite a lot … I think they actually help me boost my happiness.” Participant 160  “I used to always say walking is my therapy. It's amazing how I always felt so good, that's why I want to restart it.” Participant 691 | ***Enablement:*** Increase access to non-sedentary social activities, for example providing free admission to recreation centres or classes.  ***Modelling:*** Identify existing social connections who engage in non-sedentary hobbies or activities that may be of interest. Promote increased social outings with these friends based on shared interests and using the benefits of reducing SB as an additional motivator. | Service provision; Fiscal | 2.4 Self-monitoring of outcome(s) of behaviour  6.1 Demonstration of the behaviour  9.3 Comparative imagining of future outcomes 10.5 Social incentive |
| Reflective Motivation | Social Role/Identity | Responsibilities, such as taking care of children, pets, or household duties, keep you moving (facilitator) | “[I] come back home, make sure everyone has had dinner, tidy up, off to bed maybe ten to eleven depending, wake up [at] four or five.” Interviewer: “Okay, and you don't tend to watch TV?  Participant 589: “I don't have that luxury.”  “Well, this is my responsibility, and I love my pet, and I love her more than sitting on my comfy chair. So, she loves going outside and she is important.” Participant 008 | ***Persuasion:*** Encourage individuals to keep up with or improve habits surrounding non-SB household responsibilities, such as cooking, cleaning, dog walking, and spending time with family/child(ren).  ***Incentivization:*** Increase awareness of potential social benefits of taking on and managing responsibility, for example feeling rewarded by taking care of others, being a role model for family or peers, positively influencing others in your household, and receiving gratitude or praise. | Communication/ Marketing | 13.5 Identity associated with changed behaviour  10.5 Social incentive  10.9 Self-reward  13.1 Identification of self as role model  9.3 Comparative imagining of future outcomes  1.4 Action planning |
