# Supplementary material for: Intervention development to reduce sedentary behaviour among adults: a qualitative investigation using the Behaviour Change Wheel
Source: Int J Behav Nutr Phys Act. 2026 Apr 21;23:62. doi: 10.1186/s12966-026-01917-w (PMC13255465; doi:10.1186/s12966-026-01917-w)
Supplement: Supplementary file 4 — Supplementary Material 4 [file 12966_2026_1917_MOESM4_ESM.docx]

**
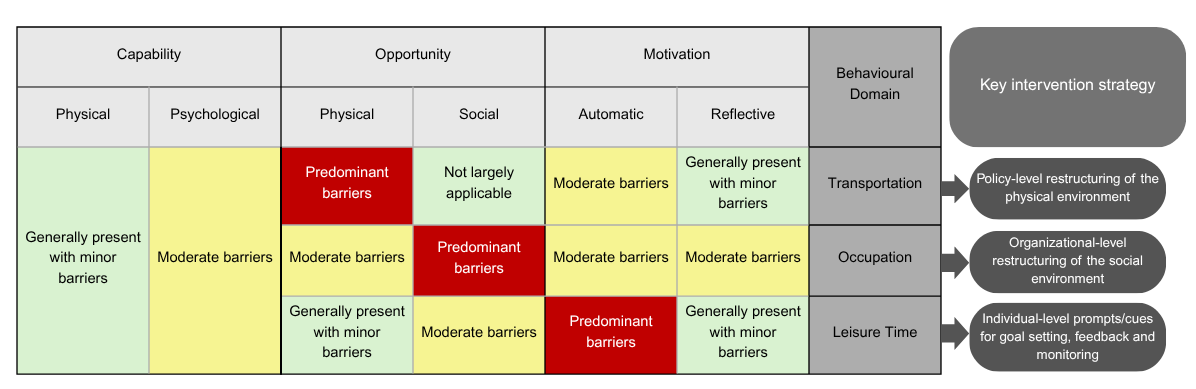
Appendix 4: Summary of behavioural determinants of reducing prolonged sedentary behaviour mapped to the COM-B model of behaviour, domains of sedentary behaviour, and resultant recommendations for intervention development**
